# Resveratrol Supplementation Prevents Hypertension in Hypertensive Pregnant Rats by Increasing Sodium Excretion and Serum Nitric Oxide Level

**DOI:** 10.1155/2020/4154010

**Published:** 2020-01-21

**Authors:** Xiaowen Jia, Rong Zhang, Jinzhu Guo, Hua Yue, Qiuxia Liu, Lipei Guo, Qunchang Zhang

**Affiliations:** Department of Gynecology and Obstetrics, XiDian Group Hospital, Xi'an, Shaanxi 710077, China

## Abstract

**Background:**

Pregnancy-induced hypertension (PIH) remains a major cause of morbidity and mortality in pregnancy worldwide. This study was designed to study the blood pressure-lowering effect of resveratrol (RES) in a salt-induced hypertensive pregnant rat model.

**Methods:**

Forty female Sprague Dawley (SD) rats were randomized into 4 groups: Normal Preg (0.9% salt diet), Normal Preg + RES (0.9% salt diet plus daily oral RES for 4 weeks), Salt Preg (8% salt diet), and Salt Preg + RES (8% salt diet plus daily oral RES for 4 weeks). Noninvasive blood pressure was recorded on gestational days 7 and 14. On the gestational day 19, foetuses were weighed, and blood and urine samples were harvested for electrolytes and biochemical assays.

**Results:**

RES significantly reduced SBP, DBP, and MAP on gestational days 7 and 14 in the Salt Preg + RES group compared to the Salt Preg group (all *P* < 0.05). Compared to the Salt Preg group, the foetal weight, serum NO level, urinary sodium, and 24 hour urine volume were significantly increased in the Salt Preg + RES group (all *P* < 0.05). Compared to the Salt Preg group, the foetal weight, serum NO level, urinary sodium, and 24 hour urine volume were significantly increased in the Salt Preg + RES group (all *P* < 0.05). Compared to the Salt Preg group, the foetal weight, serum NO level, urinary sodium, and 24 hour urine volume were significantly increased in the Salt Preg + RES group (all

**Conclusions:**

RES decreases blood pressure in a hypertensive pregnant rat model. Increasing sodium excretion and serum nitric oxide level might be, at least part of, the underlying mechanisms.

## 1. Introduction

Pregnancy-induced hypertension (PIH) complicates 6–10% of pregnancies [1]. It remains a major cause of morbidity and mortality in pregnancy worldwide, with the increased risk of renal failure, pulmonary edema, and stroke for mothers, as well as intrauterine growth restriction (IUGR), prematurity, and death for fetus [2].

It has been reported that endothelial dysfunction, oxidative stress, inflammatory responses, the renin-angiotensin system (RAS) activation, defective synthesis of nitric oxide (NO), and dysregulation of hydrogen sulfide (H_2_S) producing enzymes contribute to maternal hypertension [3–6]. However, the underlying mechanisms involved in PIH are still not completely understood.

Resveratrol (3,5,4-trihydroxystilbene, RES) is a natural polyphenolic compound found in various plants species, including grapes, berries, and peanuts [7]. Numerous studies have demonstrated the diverse biologic effects of RES, such as antioxidative, anti-inflammatory, antiviral, and antiplatelet aggregation activities [8–11]. Other studies reported it could modulate the cell functions, signal transduction, and gene expression [12]. Some recent studies demonstrated that RES could increase sodium excretion [13] and the release of NO from endothelial cells [14], as well as protect against the development of general hypertension in rat models [15–17]. However, the effect of RES on NO synthesis and sodium excretion in PIH is still unclear.

Therefore, the present research was designed to study the effect of RES on blood pressure regulation in a hypertensive pregnant rat model. Our results suggested that RES might be a potential blood pressure lowering (BPL) agent for PIH treatment.

## 2. Methods

### 2.1. Animals

A total of 20 male Sprague Dawley (SD) rats weighing between 190 and 210 g 9- to 10-week old and 40 female SD rats weighing between 180 and 200 g 9- to 10-week old were purchased from the Animal Center of Xi'an Jiaotong University (Xi'an, China). Rats were kept in sterile cages under standard laboratory conditions. All animals were allowed free access to tap water and standard rodent diet. All experiments were performed in accordance with the “National Institutes of Health Guidelines on the Use of Laboratory Animals” and approved by the Ethics Committee, Xi'an Jiaotong University Health Science Center.

### 2.2. Reagents

Resveratrol was purchased from Sigma Chemical (USA). Dimethyl sulfoxide (DMSO) and RPMI-1640 were from Xi'an Sobeo Pharmtech Co., Ltd. (Xi'an, China). The RES was dissolved in DMSO and then diluted to 5 mg/mL in RPMI-1640.

### 2.3. Experimental Design

Forty female rats were randomized into four groups (10 rats/group)s: Normal Preg: the rats were fed with normal salt chow (0.9% NaCl) for 10 weeks; Normal Preg + RES: rats were fed with the same feeding regimens as the Normal Preg group, plus daily oral RES (250 mg/kg/day, by intragastric gavage) for 4 weeks (from the 7th week till the 10th week); Salt Preg: the rats were fed with high-salt chow (8% NaCl) [18] for 10 weeks; Salt Preg + RES: rats were fed with the same feeding regimens as the Salt Preg group, plus daily oral RES (250 mg/kg/day, by intragastric gavage) for 4 weeks (from the 7^th^ week till the 10^th^ week). We choose high salt intake to induce the PIH model due to its noninvasiveness.

For these female rats, the oestrous cycle was monitored by the vaginal smear method from the 7th week [19]. Each female rat on the proestrous phase was separately mated with male rat overnight. Mating was confirmed by the presence of sperm in a vaginal smear on the next day, and this day was considered as day 1 of pregnancy.

### 2.4. Noninvasive Blood Pressure Measurement

Systolic blood pressure (SBP), diastolic blood pressure (DBP), and mean arterial blood pressure (MAP) (mmHg) were measured before mating and on gestational days 7 and 14 using a tail-cuff blood pressure instrument (Kent Scientific, Torrington, CT, USA).

### 2.5. Euthanasia and Foetal Weight Measurement

On the gestational day 19, the rats were euthanized by ether and received laparotomy. The blood samples were collected from the suprahepatic segment of inferior vena cava using a cannula for analysis, and the foetuses were isolated and weighed.

### 2.6. Serum and Urine Samples Collection

Blood samples were centrifuged at 4000 r/min at 4C for 3 min, and serum was taken and stored at –20°C for measurement of the serum electrolytes, urea, creatinine, and NO levels.

Twelve-hour urine samples were individually collected in a metabolic cage on the gestational day 18 and preserved with toluene. Urine samples were used for measurement of urinary electrolytes and protein levels, as well as urine volume.

### 2.7. Measurement of Serum Electrolytes, Urea, Creatinine, and NO Levels

Serum levels of sodium, potassium, urea, creatinine, and NO were measured by an automated chemistry analyzer (Shenzhen Kaguwi Imp & Exp Co., Ltd., Shenzhen, China) using commercial diagnostic kits following the manufacturer's instructions (Jilin Painuo Biological Technology, Ltd., Jilin, China).

### 2.8. Measurement of Urinary Electrolytes and Protein Levels

Urinary levels of sodium and potassium were measured using the same method described above for serum electrolytes. Urinary protein concentration was determined by Bradford's method (Bio-Rad protein assay, Kidlington, UK) [20].

### 2.9. Statistical Analysis

Data were presented as mean ± SD. Statistical differences were determined by using SPSS 24.0 software. As a first step, analysis of variance of factorial design was performed to check for any interaction between salt concentration and RES on blood pressure, foetal weight, and other biochemical indexes. If an interaction was ruled out, the pooled analysis remained the primary analysis. If an interaction could not be ruled out, then the effect of RES in different salt concentration subgroups would be considered by the *t*-test or the nonparametric test. Differences were considered statistically significant if the *P* value was <0.05.

## 3. Results

### 3.1. Blood Pressure

SBP, DBP, and MAP were measured before mating and on gestational days 7 and 14 using a noninvasive tail-cuff method. An interaction existed between salt concentration and RES with respect to SBP, DBP, and MAP (all *P* < 0.001). In the 10th week, compared to the Salt Preg + RES group, all three parameters were slightly increased in the Salt Preg group. However, there was no statistical difference (*P* > 0.05, data not shown). On gestational days 7 and 14, compared to the Salt Preg group, all three parameters were obviously decreased in the Salt Preg + RES group (all *P* < 0.001), but there was no statistical difference in the three parameters between group Normal Preg + RES and group Normal Preg (all *P* > 0.05). It was declared that RES has an antihypertensive effect with high concentration of salt, and it may not have antihypertensive effect if it is used for pregnancy hypertension with normal salt intake (Table 1).

### 3.2. Foetal Weight Measurement

**Figure 1:**
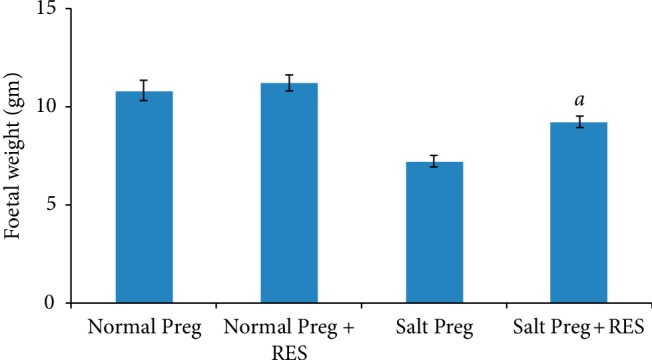
Foetal weight in hypertensive pregnant rats following RES supplementation. ^*a*^*P* < 0.05 vs. Salt Preg.

On the gestational day 19, the foetuses were isolated and weighed. The interaction existed between salt concentration and RES with respect to foetal weight (*P* < 0.05). Compared to the Salt Preg group, the foetal weight was increased in the Salt Preg + RES group (*P* < 0.05), and there was no statistical difference in foetal weight between the Normal Preg + RES group and Normal Preg group (*P* > 0.05) (Figure 1).

### 3.3. Measurement of Serum Electrolytes, Urea, Creatinine, and NO Levels

**Figure 2:**
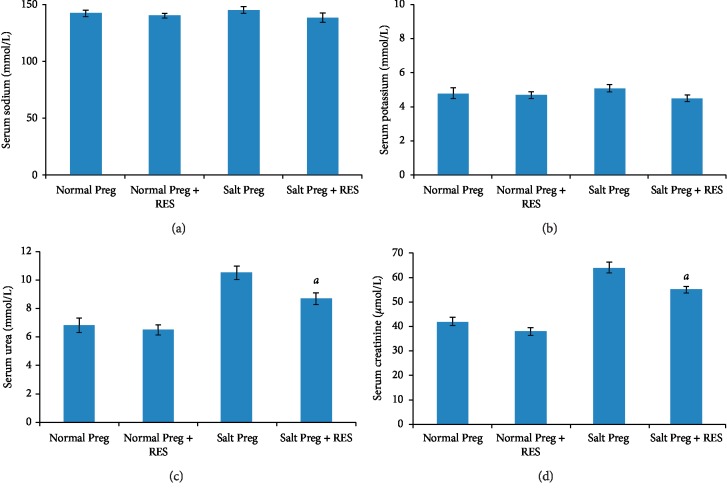
Serum levels of sodium (a), potassium (b), urea (c), and creatinine (d) in hypertensive pregnant rats following RES supplementation. ^*a*^*P* < 0.05 vs. Salt Preg.

**Figure 3:**
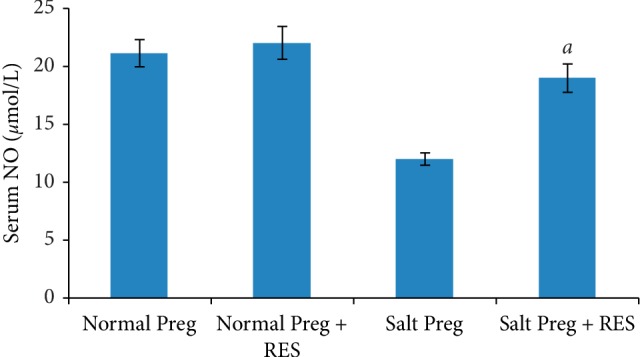
Serum level of NO in hypertensive pregnant rats following RES supplementation. ^*a*^*P* < 0.05 vs. Salt Preg.

The interaction existed between salt concentration and RES with respect to serum sodium, potassium, urea, creatinine, and NO levels (all *P* < 0.05). There was no statistical difference in serum sodium and potassium between the Salt Preg + RES group and Salt Preg group or between the Normal Preg + RES group and Normal Preg group (all *P* > 0.05) (Figures 2(a) and 2(b)). The levels of serum urea and serum creatinine were significantly higher in the Salt Preg group compared to the Salt Preg + RES group (all *P* < 0.05), and there was no statistical difference in serum urea and serum creatinine between the Normal Preg + RES group and Normal Preg group (*P* > 0.05) (Figures 2(c) and 2(d)). For the level of serum NO, it was significantly lower in the Salt Preg group than the Salt Preg + RES group (*P* < 0.05), and there was no statistical difference in serum NO between the Normal Preg + RES group and Normal Preg group (*P* > 0.05) (Figure 3).

### 3.4. Measurement of Urinary Electrolytes and Protein Levels

**Figure 4:**
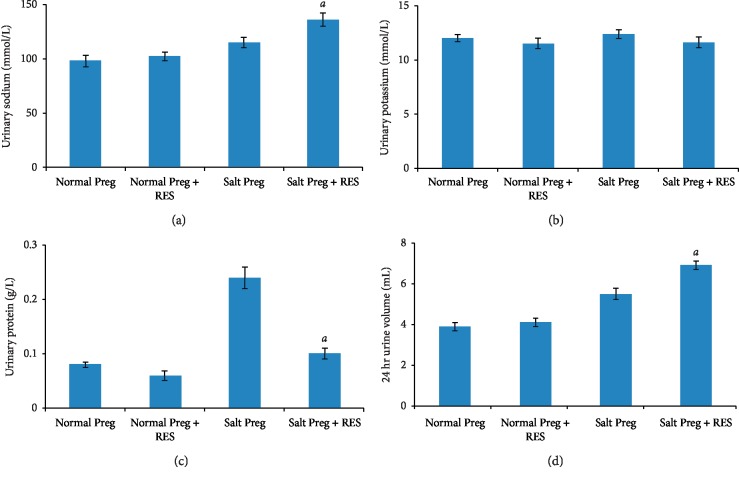
Urinary levels of sodium (a), potassium (b), and protein (c), as well as 24 h urine volume in hypertensive pregnant rats following RES supplementation. ^*a*^*P* < 0.05 vs. Salt Preg.

The interaction existed between salt concentration and RES with respect to urinary sodium, potassium, and protein levels (all *P* < 0.05). There was no statistical difference in urinary potassium between the Salt Preg + RES group and Salt Preg group or between Normal Preg + RES group and Normal Preg group (all *P* > 0.05). However, the urinary sodium was significantly increased in the Salt Preg + RES group compared to the Salt Preg group (*P* < 0.05), and there was no statistical difference in urinary sodium between the Normal Preg + RES group and Normal Preg group (*P* > 0.05) (Figures 4(a) and 4(b)). For the urinary protein level, it was obviously higher (*P* < 0.05) in the Salt Preg group than the Salt Preg + RES group, and there was no statistical difference in urinary protein between the Normal Preg + RES group and Normal Preg group (*P* > 0.05) (Figure 4(c)).

### 3.5. Twenty-Four-Hour Urine Volume

The interaction existed between salt concentration and RES with respect to 24-hour urine volume (*P* < 0.05). The 24-hour urine volume was significantly higher in the Salt Preg + RES group compared to the Salt Preg group, and there was no statistical difference in 24-hour urine volume between the Normal Preg + RES group and Normal Preg group (*P* > 0.05) (Figure 4(d)).

## 4. Discussion

The effect of RES on blood pressure regulation has been previously studied. A recent systematic review concluded that RES appears to have antihypertensive effects, depending on the dose and duration of treatment, and one of the important blood pressure-reducing mechanisms of RES is to increase the level of NO [21]. However, the effect of RES on PIH remains unclear. In the present study, we find that oral RES supplementation could decrease the blood pressure of high-salt diet-induced hypertensive pregnant rats compared to control animals without RES supplementation (Table 1).

Increased salt intake is an important factor in elevating the BP in human [22]. Sodium retention causes expansion of extracellular volume (ECV) and total vascular volume, which may lead to hypertension. Thus, reducing the amount of sodium in the body became an important therapeutic strategy for decreasing BP. For example, diuretics exert their hypotensive effects via increasing sodium excretion [23]. In our study, we used high-salt diet to induce hypertension in pregnant rats. Our results demonstrated that RES supplementation could significantly increase the excretion of sodium in urine without affecting the serum sodium level in a hypertensive pregnant rat model. Consistent with this, the 24 h urine volume was also significantly increased in the hypertensive pregnant rats treated with RES supplementation compared to control rats without RES treatment (all *P* < 0.05). These findings are consistent with a published report showing RES infusion increases sodium excretion while not altering the glomerular filtration rate (GFR), suggesting it may have a direct effect on renal tubular sodium handling [24].

Preeclampsia (PE) complicates about 3–5% of all pregnancies and is simply defined by the new onset of hypertension and proteinuria occurring from 20 weeks of gestation [25]. Studies had demonstrated that kidney function injury frequently occurred in PE patients even before proteinuria detection [26]. The protective effect of RES on kidney functions had been previously studied. Kitada et al. showed that, in diabetic db/db mice, RES treatment for 8 weeks (0.3% diet) resulted in decreased urinary albumin excretion [27]. Qiao et al. showed that treatment of STZ-induced diabetic SD rats with RES (20 mg/kg/day) for 4 weeks resulted in reduced serum glucose and creatinine levels [28]. Consistent with these results, we observed that the serum urea and creatinine levels, as well as the urinary protein level, were obviously increased in a rat model of pregnant hypertension. However, RES supplementation significantly reduced these parameters compared to control pregnant rats (all *P* < 0.05).

Another suggested mechanism of PIH is defective NO synthesis, which could lead to endothelial dysfunction [29]. NO-mediated activation of cGK leads to relaxation of vascular smooth muscle cells, which could cause vasodilatation and subsequent blood pressure reduction [30]. Previous studies had showed that RES could increase the production of NO in endothelial cells by upregulating the expression of endothelial NO synthase (eNOS), stimulating eNOS enzymatic activity, and preventing eNOS uncoupling [31]. In a recent study, Xu et al. found RES could also stimulate NO release in rat platelets [32]. In this study, the level of serum NO was significantly decreased in the pregnant hypertensive rats, and as expected, RES supplementation significantly increased it to a level comparable to nonhypertensive pregnant control rats. Previous studies had demonstrated that NO is also a potent natriuretic agent, playing a major role in inhibiting epithelial sodium channel (ENaC) activity in the collecting duct [33], which hinted that the different antihypertensive mechanisms of RES might result in a synergistic effect.

Having low birth weight is associated with fetal and neonatal morbidity and mortality [34]. PIH had been identified to be closely associated with low birthweight [35]. The weight of the foetal rat on the 19th day of gestation varied slightly in different reports. In our study, the foetal weights in normal salt intake groups are comparable to that reported by Arikawe et al.[18]. However, foetal weight was obviously lower in the hypertensive pregnant rats than the control pregnant rats, and RES supplementation partly reversed this trend (all *P* < 0.05). These results are in line with a previous study showing subcutaneous maternal RES treatment increases uterine artery blood flow in the pregnant ewe and increases fetal growth [36]. We hypothesized that the effect of increasing foetal weight by RES may be related to its upregulation on the serum NO level.

## 5. Conclusion

Taken together, our present study has provided the first evidence that increasing sodium excretion and serum nitric oxide level might be, at least part of, the underlying mechanisms by which RES decreases blood pressure in a hypertensive pregnant rat model. RES could be a promising candidate in the development of an effective BPL agent for PIH treatment.

However, the mechanism of PIH in humans is complex, and high salt intake or defective NO synthesis is only part of the risk factors. Therefore, further studies are needed to identify the detailed mechanisms by which RES regulated blood pressure in pregnancy for its possible application in the treatment of PIH.

## Figures and Tables

**Table 1:** Effect of RES on blood pressure in hypertensive pregnant rats.

Blood pressure		Normal Preg	Normal Preg + RES	Salt Preg	Salt Preg + RES	Salt^*∗*^ RES^1^		Normal Preg + RES vs. Normal Preg		Salt Preg + RES vs. Salt Preg	
						*F*	*P*	*t*	*P*	*t*	*P*
SBP (mmHg)	Gestational day 7	117.3 ± 3.7	120.4 ± 2.9	148.5 ± 4.9	130.1 ± 3.2	95.907	<0.001	–2.046	0.056	11.281	<0.001
	Gestational day 14	99.5 ± 4.0	97.5 ± 24.2	151.8 ± 5.1	128.5 ± 3.7	61.129	<0.001	1.122	0.277	11.684	<0.001

DBP (mmHg)	Gestational day 7	85.7 ± 3.7	86.9 ± 3.3	116.5 ± 2.6	107.5 ± 3.2	25.189	<0.001	–0.748	0.464	6.972	<0.001
	Gestational day 14	60.4 ± 4.5	57.9 ± 4.7	113.8 ± 3.4	96.9 ± 3.8	30.174	<0.001	1.237	0.232	10.516	<0.001

MAP (mmHg)	Gestational day 7	92.5 ± 4.2	94.4 ± 5.4	130.0 ± 4.9	111.4 ± 4.5	46.344	<0.001	–0.886	0.388	8.840	<0.001
	Gestational day 14	74.3 ± 4.0	77.3 ± 4.2	118.7 ± 4.6	99.4 ± 2.3	82.780	<0.001	–1.652	0.116	11.850	<0.001

Salt^*∗*^ RES^1^, interaction between salt and RES.

## Data Availability

All data generated or analysed during this study are included in this published article.

## References

[1] Kintiraki E., Papakatsika S., Kotronis G., Goulis D., Kotsis V. (2015). Pregnancy-induced hypertension. *Hormones*.

[2] Kouhkan A., Khamseh M. E., Pirjani R (2018). Obstetric and perinatal outcomes of singleton pregnancies conceived via assisted reproductive technology complicated by gestational diabetes mellitus: a prospective cohort study. *BMC Pregnancy and Childbirth*.

[3] Sacks G. P., Studena K., Sargent I. L., Redman C. W. G. (1998). Normal pregnancy and preeclampsia both produce inflammatory changes in peripheral blood leukocytes akin to those of sepsis. *American Journal of Obstetrics and Gynecology*.

[4] Zhou Y., Damsky C. H., Chiu K., Roberts J. M., Fisher S. J. (1993). Preeclampsia is associated with abnormal expression of adhesion molecules by invasive cytotrophoblasts. *Journal of Clinical Investigation*.

[5] Oludare G. O., Ilo O. J., Lamidi B. A. (2017). Effects of lipopolysaccharide and high saline intake on blood pressure, angiogenic factors and liver enzymes of pregnant rats. *Nigerian Journal of Physiological Sciences: Official Publication of the Physiological Society of Nigeria*.

[6] Wang K., Ahmad S., Cai M. (2013). Dysregulation of hydrogen sulfide producing enzyme cystathionine γ-lyase contributes to maternal hypertension and placental abnormalities in preeclampsia. *Circulation*.

[7] Koushki M., Amiri-Dashatan N., Ahmadi N., Abbaszadeh H. A., Rezaei-Tavirani M. (2018). Resveratrol: a miraculous natural compound for diseases treatment. *Food Science & Nutrition*.

[8] Robb E. L., Page M. M., Wiens B. E., Stuart J. A. (2008). Molecular mechanisms of oxidative stress resistance induced by resveratrol: specific and progressive induction of MnSOD. *Biochemical and Biophysical Research Communications*.

[9] Lanzilli G., Cottarelli A., Nicotera G., Guida S., Ravagnan G., Fuggetta M. P. (2012). Anti-inflammatory effect of resveratrol and polydatin by in vitro IL-17 modulation. *Inflammation*.

[10] Abba Y., Hassim H., Hamzah H., Noordin M. M. (2015). Antiviral activity of resveratrol against human and animal viruses. *Advances in Virology*.

[11] Toliopoulos I. K., Simos Y. V., Oikonomidis S., Karkabounas S. C. (2013). Resveratrol diminishes platelet aggregation and increases susceptibility of K562 tumor cells to natural killer cells. *Indian Journal of Biochemistry and Biophysics*.

[12] Kovacic P., Somanathan R. (2010). Multifaceted approach to resveratrol bioactivity: focus on antioxidant action, cell signaling and safety. *Oxidative Medicine and Cellular Longevity*.

[13] Gordish K. L., Beierwaltes W. H. (2016). Chronic resveratrol reverses a mild angiotensin II-induced pressor effect in a rat model. *Integrated Blood Pressure Control*.

[14] Toaldo I. M., Van Camp J., Gonzales G. B. (2016). Resveratrol improves TNF-α-induced endothelial dysfunction in a coculture model of a Caco-2 with an endothelial cell line. *The Journal of Nutritional Biochemistry*.

[15] Sarkar O., Li Y., Anand-Srivastava M. B. (2019). Resveratrol prevents the development of high blood pressure in spontaneously hypertensive rats through the inhibition of enhanced expression of Gi*α* proteins. *Canadian Journal of Physiology and Pharmacology*.

[16] Yeh T. C., Shin C. S., Chen H. H (2018). Resveratrol regulates blood pressure by enhancing AMPK signaling to downregulate a Rac1-derived NADPH oxidase in the central nervous system. *Journal of Applied Physiology*.

[17] Fabricio V., Oishi J. C., Biffe B. G (2017). Resveratrol treatment normalizes the endothelial function and blood pressure in ovariectomized rats. *Arquivos Brasileiros de Cardiologia*.

[18] Arikawe A. P., Udenze I. C., Olusanya A. W., Akinnibosun O. A., Dike I., Duru B. N. (2019). L-arginine supplementation lowers blood pressure, protein excretion and plasma lipid profile in experimental salt-induced hypertension in pregnancy: relevance to preeclampsia. *Pathophysiology*.

[19] Marcondes F. K., Bianchi F. J., Tanno A. P. (2002). Determination of the estrous cycle phases of rats: some helpful considerations. *Brazilian Journal of Biology*.

[20] Salas S. P., Giacaman A., Vío C. P. (2003). Pregnant rats with 5/6 nephrectomy have normal volume expansion despite lower renin and kallikrein. *Hypertension*.

[21] Dorri Mashhadi F., Salari R., Ghorbanzadeh H. (2019). The effect of resveratrol dose and duration of treatment on blood pressure in patients with cardiovascular disorders: a systematic review. *Current Drug Discovery Technologies*.

[22] Ivy J. R., Bailey M. A. (2014). Pressure natriuresis and the renal control of arterial blood pressure. *The Journal of Physiology*.

[23] Garfinkle M. A. (2017). Salt and essential hypertension: pathophysiology and implications for treatment. *Journal of the American Society of Hypertension*.

[24] Gordish K. L., Beierwaltes W. H. (2014). Sustained resveratrol infusion increases natriuresis independent of renal vasodilation. *Physiological Reports*.

[25] Moghaddas Sani H., Zununi Vahed S., Ardalan M. (2019). Preeclampsia: a close look at renal dysfunction. *Biomedicine & Pharmacotherapy*.

[26] Armaly Z., Jadaon J. E., Jabbour A., Abassi Z. A. (2018). Preeclampsia: novel mechanisms and potential therapeutic approaches. *Frontiers in Physiology*.

[27] Kitada M., Kume S., Imaizumi N., Koya D. (2011). Resveratrol improves oxidative stress and protects against diabetic nephropathy through normalization of Mn-SOD dysfunction in AMPK/SIRT1-independent pathway. *Diabetes*.

[28] Qiao Y., Gao K., Wang Y., Wang X., Cui B. (2017). Resveratrol ameliorates diabetic nephropathy in rats through negative regulation of the p38 MAPK/TGF-*β*1 pathway. *Experimental and Therapeutic Medicine*.

[29] Shashar M., Zubkov A., Chernichovski T. (2019). Profound decrease in glomerular arginine transport by CAT (cationic amino acid transporter)-1 contributes to the FLT-1 (FMS-Like tyrosine kinase 1) induced preeclampsia in the pregnant mice. *Hypertension*.

[30] Li Q., Youn J.-Y., Cai H. (2015). Mechanisms and consequences of endothelial nitric oxide synthase dysfunction in hypertension. *Journal of Hypertension*.

[31] Li H., Xia N., Hasselwander S., Daiber A. (2019). Resveratrol and vascular function. *International Journal of Molecular Sciences*.

[32] Xu M., Xue W., Ma Z., Bai J., Wu S. (2016). Resveratrol reduces the incidence of portal vein system thrombosis after splenectomy in a rat fibrosis model. *Oxidative Medicine and Cellular Longevity*.

[33] West C. A., Sasser J. M., Baylis C. (2016). The enigma of continual plasma volume expansion in pregnancy: critical role of the renin-angiotensin-aldosterone system. *American Journal of Physiology-Renal Physiology*.

[34] Chiarotti F., Castignani A. M., Puopolo M., Menniti-Ippolito F., Minniti De Simeonibus E., Di Paolo A. (2001). Effects of socio-environmental factors on neurocognitive performance in premature or low-birth weight preschoolers. *Annali dell’Istituto Superiore di Sanità*.

[35] Lake E. A., Olana Fite R. (2019). Low birth weight and its associated factors among newborns delivered at wolaita sodo university teaching and referral hospital, southern Ethiopia, 2018. *International Journal of Pediatrics*.

[36] Darby J. R. T., Saini B. S., Soo J. Y (2019). Subcutaneous maternal resveratrol treatment increases uterine artery blood flow in the pregnant Ewe and increases fetal but not cardiac growth. *The Journal of Physiology*.

